# *Deverra triradiata* Hochst. ex Boiss. from the Northern Region of Saudi Arabia: Essential Oil Profiling, Plant Extracts and Biological Activities

**DOI:** 10.3390/plants11121543

**Published:** 2022-06-09

**Authors:** Arbi Guetat, Abdelrahman T. Abdelwahab, Yassine Yahia, Wafa Rhimi, A. Khuzaim Alzahrani, Abdennacer Boulila, Claudia Cafarchia, Mohamed Boussaid

**Affiliations:** 1 Department of Biological Sciences, College of Sciences, Northern Border University, Arar 92341, Saudi Arabia; abdelrhman.talha@nbu.edu.sa; 2 Laboratory of Nanobiotechnology and Valorisation of Medicinal Phytoresources, National Institute of Applied Science and Technology, University of Carthage, Tunis 1080, Tunisia; mohamed.boussaid@insat.rnu.tn; 3 Department of Botany and Microbiology, Faculty of Science, Al-Azhar University, Cairo 4293073, Egypt; 4 Laboratoire d’Aridoculture et Cultures Oasiennes, Institut des Régions Arides de Médenine, Médenine 4119, Tunisia; yahia.yassine@gmail.com; 5 Faculté des Sciences de Bizerte, Zarzouna, Université de Carthage, Carthage 7021, Tunisia; wafa_rhimi@hotmail.com; 6 Dipartimento di Medicina Veterinaria, Università degli Studi di Bari, 70010 Valenzano, Italy; claudia.cafarchia@uniba.it; 7 Faculty of Applied Medical Sciences, Northern Border University, Arar 92341, Saudi Arabia; ahmed.aldousi@nbu.edu.sa; 8 Laboratory of Natural Substances LR10INRAP02, National Institute of Research and Physico-Chemical Analyses, Biotechnopole of Sidi Thabet, Ariana 2020, Tunisia; abdennacer.boulila@inrap.rnrt.tn

**Keywords:** essential oils, plant extracts, *Deverra triradiata*, Saudi Arabia, biological activities

## Abstract

*Devrra triradiata* Hochst. ex Boiss is an occasional plant species in the Northern region of Saudi Arabia. The shrub is favored on sandy desert wadis, gypsaceous substrate, and sandy gravel desert. In folk medicine, the plant is used for many purposes; to relieve stomach pains, against intestinal parasites, and for the regulation of menstruation. The present study describes the chemical composition of the essential oils (EOs) of different plant parts of *D. triradiata*. In vivo and in vitro biological activities of plant extracts and essential oils were also studied. Phenylpropanoids, elemicin (flowers: 100%), dillapiole (Stems: 82.33%; and seeds: 82.61%), and apiol (roots: 72.16%) were identified as the major compounds. The highest antioxidant activity was recorded for the EOs of roots and stems (IC_50_ = 0.282 µg/mL and 0.706 µg/mL, respectively). For plant extracts, ethyl acetate showed the highest antioxidant activities (IC_50_ = 2.47 and 3.18 µg/mL). EOs showed high antifungal activity against yeasts with low azole susceptibilities (i.e., Malassezia spp. and Candida krusei). The MIC values of EOs ranged between 3.4 mg/mL and 56.4 mg/mL. The obtained results also showed phytotoxic potential of plant extracts both on the germination features of *Triticum aestivum* seeds and the vegetative growth of seedlings.

## 1. Introduction

The Apiaceae family (Umbelliferae), known under the name of the carrot or parsley family, is one of the most important families of flowering plants. This family encompasses aromatic plants of economic importance employed in foodstuffs, beverages, perfumery, pharmaceuticals, and cosmetics [1]. Species of this family include lianas, herbs, shrubs, or trees [2]. This family is nearly cosmopolitan, being diverse from tropical to temperate regions [3] and consisting of some 455 genera and more than 3700 species widely distributed across temperate regions, especially in Central Asia [4,5]. South-west Asia is an area of considerable diversity and endemism in this family [6].

In Saudi Arabia, the flora is one of the richest in biodiversity in the Arabian Peninsula and comprises very important genetic resources of crop and medicinal plants [7]. The flora of the country consists of more than 140 families, 835 genera, and 2250 species. It was estimated that the flora of Saudi Arabia is diverse and has a great number of medicinal species, which is expected to exceed 1200 (over 50%) [7] and about 471 species (20%) according to Aati et al. [8]. Among others, Asteraceae, Fabaceae, Lamiaceae, and Apiaceae are the most common families in the flora of Saudi Arabia [7,9,10]. Collenette [7] reported that Apiaceae is represented by about 24 genera and more than 45 species in the flora. However, Pimenov and Lenov [6] signaled that the family is present with 26 genera and 52 species.

In the Northern Region of the country (125,000 sq.km), two genera of the Apiaceae family are common: The genus *Deverra* with two species (*Deverra tortuosa* and *Deverra triradiata*) and the genus *Ducrosia* with two species (*Ducrosia anethifolia* and *Ducrosia flabellifolia*). These species are widespread in the Al Widyian region (Wadis), especially the Wadi Arar’s tributaries. Taxa belonging to the genus *Deverra* in Saudi Arabia are poorly studied [10]. To the best of our knowledge, phytochemical investigation of *D. triradiata* (ex- *Pituranthos triradiatus* (Boiss.) Asch. & Schweinf) in Saudi Arabia is completely absent. The species is an occasional shrub in the Northern region of Saudi Arabia along stony plains and wadis (Al Widyian region). Once compared to *D. tortuosa*, *D. triradiata* is occasional and less frequent, and the two taxa are found in sympatric association. The species is favored on sandy desert wadis, gypsaceous substrate, and sandy gravel desert of Iraq, Egypt, Sinai, and Arabia [11]. The plant is in the bloom stage during the summer season from July until November (personal observation) [11].

The botanical description is reported by Chrtek et al. [12] and confirmed by Boulos [13]: *D. triradiata* Hochst. ex Borns is a perennial yellow-green plant, reaching 35–100 cm in height. The species is a glabrous shrub with erect stems, juncaceous, sparsely alternately branched, and stout branches. Cauline leaves are strongly reduced to short ovate-triangular sheaths, sometimes with remains of lamina. Umbels (2,3, or 6 flowers) are with glabrous unequal long rays. Bracts are early caducous; pedicels are unequal (2 mm long) and whitish shortly hairy; bracteoles are caducous. Fruits are narrowly ovoid or oblong (with 3 to 5 mm long) and densely hairy, hairs (±0.5 mm long) and the mericarp are three times longer than broad.

In Saudi Arabia, the species is known under the vernacular name of “Haza” or “Sousse”. According to Halim et al. [14], the plant is used locally by the Bedouin population against stomach pain, intestinal parasites, haematuria, blood cough, and in the regulation of menstruation. Guetat (2022) [15], reported that the species is described to be a plant of interest in the restoration of sandy coastal areas [16]. According the same author [15], the ethnopharmacologial uses of the plant indicated that *D. triradiata* is used to relieve stomach pains, against intestinal parasites, and the regulation of menstruation [14,17,18,19,20]. A strong photodermatitis induced by the twigs of plants during the collection and furanocoumarins of the plant were reported to be the cause [15,17,18,19,20]. Bergapten is a linear furocoumarins, also known as 5-methoxypsoralen, with a wide range of pharmacological effects, including neuroprotection, organ protection, anticancer, anti-inflammatory, antimicrobial, and antidiabetes effects [21]. Furthermore, the compound was isolated many times (Ashkenazy and his collaborators) from *D. triradiata* [15]. In the last few decades, multidrug-resistant (MDR) organisms have increasingly become a serious issue in clinical practices and the emergence of drug resistance in pathogens is becoming progressively more common [22]. The term antimicrobial resistance (AMR) is used and referring to the development of resistance by parasites, protozoa fungi, viruses, and MDR bacteria. Many fungi are parasites for plants, animals, human, and other fungi. Plant pathogenic fungi are able to cause extensive damage and losses to agriculture and forestry and can cause serious diseases in humans, including aspergillosis, candidosis, coccidioidomycosis, etc. [23]. The emergence of antifungal resistance (AFR) has urged researchers to explore therapeutic alternatives, one of which includes the use of natural plant products such as EOs.

Studies focusing on essential oil profiling and bioactivities of phytochemicals from the species are sparse. This study bridges this gap as it seeks (i) to profile the essential oils of roots, seeds, stems, and flowers of Saudi Arabian *D. triradiata* and (ii) to study the antioxidant, antifungal, and allelopathic activities of EOs and plant extracts.

## 2. Material and Methods

### 2.1. Plant Material

In September 2018, *D. triradiata* Hochst. ex Boiss. was collected from the wild. The site of the collection is situated about 50 km to the East of Arar city (Northern region of Saudi Arabia) in Wadi Aboulkour (30° 44′ 9.816″; 41° 27′ 8.928″), a tributary of Wady Arar. The plant specimen was identified in the Department of Biology, College of Sciences, Northern Border University. A voucher specimen was deposited (Dtr891) in the herbarium of the College of Sciences.

### 2.2. Isolation of Essential Oil and Crude Collection

Using Clevenger-type apparatus, the essential oils (EOs) were extracted from plant materials (100 g) and dried at laboratory room temperature (25 °C). Flowers, stems, roots, and seeds were used for EOs extraction by hydrodistillation for 4 h. Anhydrous sodium sulfate was used for drying the obtained EOs. Oil yield was estimated based on the 100 g dry weight of plant material and stored in dark vials (4 °C) for further analysis.

For crude collection, powder of different plant parts (100 g) was macerated (7 days) in a glass container with organic solvents (petroleum ether, EtOAc, and MeOH) at room temperature (RT: 25 °C). Plant material was deposited in extraction thimbles and covered by an organic solvent (500 mL). One week later, plant extracts were filtered through Whatman paper and the obtained crudes were leaded. Petroleum ether lead to a crude extract of 800 mg and 4.43 g, respectively, for roots and aerial parts, ethyl acetate lead to a crude extract of 300 mg and 1.75 g, respectively, for roots and aerial parts, and finally, methanol lead to a crude extract of 1.93 g and 8.52 g, respectively, for roots and aerial parts.

### 2.3. Chemicals

All solvents and chemicals are of analytical grade. DPPH (2,2-diphenyl-2-picrylhydrazyl) and ABTS (2,2-azino-bis-(3-ethylbenzothiazoline-6-sulphonic acid)) were obtained from Sigma Aldrich, (Burlington, MA, USA).

### 2.4. Gas Chromatography

For GC analyses, a 0.2 μL sample of essential oil (diluted in dichloromethane: 1:100) was used. The analyses were carried out using Agilent gas chromatography (HP7890 GC). The apparatus was equipped with a flame ionization detector (FID) and an HP-5 fused silica column (30 m × 0.32 mm, 0.25 μm film thickness). As a carrier gas, nitrogen was used for the analysis of EOs samples. The injector and detector temperatures were 210 °C and 230 °C, respectively. The column oven temperature varied from 60 °C to 220 °C, with an increasing rate of 3 °C/min.

### 2.5. GC-MS Analyses

Analyses of EOs samples (0.2 μL) were performed on an Agilent mass spectrometer (Model HP 5975 C). The apparatus was coupled with an Agilent gas chromatograph HP-5MS capillary column (30 m × 250 µm coated with 5% phenyl methyl silicone, 95% dimethylpolysiloxane, and 0.25 µm film thickness). Helium was used as a carrier gas, and the flow rate was fixed to 0.8 mL/min. The oven temperature was initially programmed to vary from 60 to 220 °C with an increasing rate of 4 °C/min, and the transfer line temperature was 230 °C.

### 2.6. Identification of Components

The profiling of EOs components was performed according to the retention index (RI). The Mass Spectroscopy library (NIST/Wiley) and available literature [24] were referred to for recorded mass spectra. Two Databases were used for the identification of the EOs components synonyms (Database\NIST05a.L Minimum Quality: 90; Database\Wiley7Nist05.L). Common names are cited according to PubChem (https://pubchem.ncbi.nlm.nih.gov: last accessed date on 25 May 2022) and NISTBOOK (https://webbook.nist: last accessed date on 25 May 2022).

### 2.7. Antioxidant Activities 

#### 2.7.1. DPPH Radical Scavenging Assays

The used method was described by Guetat et al. [10], as described previously by Brand et al. [25], with slight modifications. Plant extracts and EOs samples extracts were prepared in methanol. Subsequently, 60 µL of oil/extracts (at different concentrations) were added to 2940 µL of the methanolic DPPH solution (100 µM). The mixture was conserved in the dark for 30 min and a spectrophotometer (V-630 UV-Vis Spectrophotometer from Jasco) was used to perform the analysis at 517 nm against the blank sample. As a negative control, methanol solution was used, and DPPH solution was referred to as a positive control. The radical-scavenging activity was calculated using the following equation:

DPPH_ScA_ (%) = [(AB–AA)/AB] × 100 (1)

where DPPH_ScA_ is the percentage of DPPH inhibition; AB and AA are, respectively, the optical density (OD) values of the positive control and the OD of the test sample. IC_50_ values were presented as results, where IC_50_ means the concentration of the antioxidant sufficient to scavenge 50% of DPPH present in the test solution. The experiment was replicated three times and IC_50_ values were reported as means ±SD.

#### 2.7.2. ABTS Radical Cation Decolorization Assay 

The method used was described by Guetat et al. [10] and Dorman and Hiltunen [26]; the total radical-scavenging capacity was evaluated by the ability of the sample to scavenge the ABTS radical (ABTS•+). The ABTS•+ solution was prepared by mixing 7 mM ABTS and 2.45 mM K_2_S_2_O_8_, and the mixture was stored in darkness at RT for 12 h [10,27,28]. ABTS•+solution was diluted in order to obtain an absorbance of 0.7 ± 0.01 at 734 nm. 60 µL of diluted oil and the extract was added to 2940 µL ABTS•+ solution and the absorbance at 734 nm was then measured. The blank consisted of 60 µL of the solvent added to 2940 µL of the ABTS•+ solution. The absorbance was recorded at RT 10 min after the addition of the antioxidant. The experiment was performed in triplicate.

### 2.8. Minimal Inhibitory Concentration (MIC) and Minimal Fungicidal (MFC) Concentration 

In the present study, Table 1 shows the used yeast strains (5 *Candida* spp. strains and 6 *Malassezia* spp. Strains). A non-selective isolation medium (Sabouraud Dextrose Agar: SAB) was used for *Candida* spp. isolation and cultivation. However, Dixon Agar (DXA) was used for *Malassezia* spp. strains. Formerly, fungal strains were suspended in 5 mL of sterile saline and vortexed for 15 s. Saline solution (0.85% NaCl) was added until obtaining 0.5 and 2.04 values in the McFarland scale, respectively, for *Candida* spp. and *Malassezia* spp.

For *Candida* spp., EOs (3, 6 µL, 12, 24, and 48 µL/mL) were prepared in RPMI 1640 medium (pH 7). However, for *Malassezia* spp., natural products (EOs with the same concentrations used for Candida spp.) were prepared in SAB + 1% Tween 80. Aliquots of EOs samples (100 µL) and selected *Candida* and *Malassezia* species (100 µL) were bestowed into 96-well plates (Orange Scientific, Braine-l’Alleud). As a control, samples of EOs and yeast-free strains were also conferred. The 96-well plates were incubated at 37 °C for 48 h to 72 h. The MIC values were determined after the visualization of the resultant plate. MIC corresponds to the lower concentration of the antifungal agent in which no visible growth of the fungus was observed, compared to the control (strains grown without oil). The number of viable cells was assessed by the determination of the number of colonies forming units (CFUs) through several dilutions. The MFC values were defined as the antifungal agent concentration in which no CFUs were counted. The experiment was repeated in triplicate in three independent replications.

### 2.9. D. triradiata and Phytotoxic Potential of the Plant Extracts

The allelopathic activity of the roots and aerial parts extracts of *D. triradiata* was evaluated against wheat (*Triticum aestivum* L.) seeds germination and seedlings growth. Before starting the germination experiments, seeds were sterilized with 0.3% NaOCl for 5 min. Five replicates (100 seeds) were prepared for each concentration of the plant extracts. To examine the allelopathic effect of the extracts, various concentrations (0.2, 0.4, 0.6, 0.8, and 1 mg/mL) were prepared in organic solvents (petroleum ether, ethyl acetate, and methanol). Prior to the germination experiment, plant extract (5 mL) was added to Petri dishes (9 cm) lined with a filter paper Whatman No. 1 and air-dried at laboratory room temperature (25 °C) for 24 h. Then, 5 mL of distilled water was added to each Petri dish. The negative control consists of germinated seeds using distilled water and without plant extracts. The Petri dishes were wrapped with Parafilm tape and incubated in the darkness at 25 °C for 1 week [10]. Hypocotyl lengths, root lengths, fresh seedlings weight, and dry biomass weights per Petri dish were determined to evaluate the allelopathic activity of the plant extracts. The inhibitory or stimulatory effects were calculated using the following equation [28]:

PytPot *_D. triradiata_* (%) = [(PEE–CE)/CE] × 100 (2)

where PEE: the effect of plant extract and CE: the null effect of the negative control.

### 2.10. Statistical Analysis

All results of biological activities (antioxidant, antifungal, and allelopathic) were expressed as mean values ±SD, and the experiments were replicated three times. ANOVA procedure was used to assess the quantitative differences. Duncan’s multiple range test was referred to test the significance (*p* < 0.05) of the mean difference.

## 3. Results and Discussion 

### 3.1. EOs Profiling 

**Figure 1:**
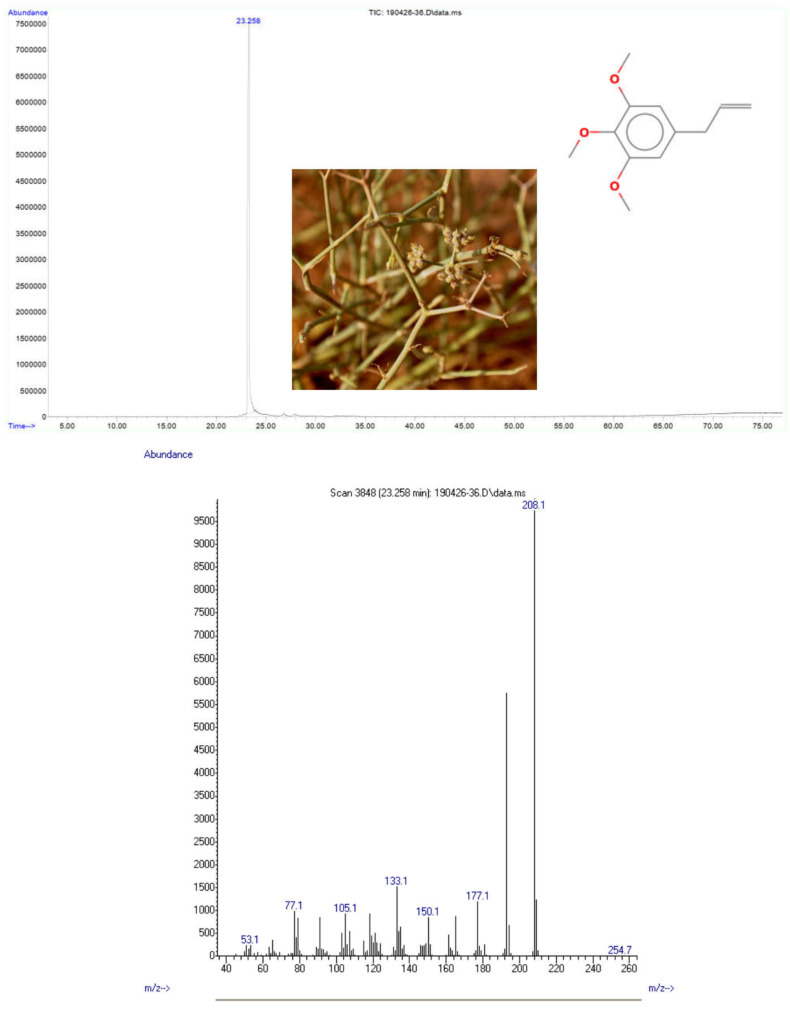
Mass spectra of elemicin as the unique pure compound isolated from flowers’ EOs of *D. triradiata*.

The obtained EOs from stems, seeds, and flowers were a light yellow to green color. EOs yields of these plant parts varied from 0.53, 0.42, and 0.36% (g EOs/100 g of dry plant material), respectively, for stems, seeds, and flowers. However, the oil of the roots was more viscous, colored dark golden and with the lowest yield (0.045% g of EOs/100 g of dry plant material). Nine phytochemicals representing 100% of the total oil were identified as constituents of the EOs by combined GC and GC/MS analyses (Table 2). The aromatic phenylpropanoid elemicin was identified as the unique compound of flowers’ EOs (100%). However, this compound is present in the EOs of seeds with a low percentage (3.12%), and it is totally absent in the EOs of stems and roots (Table 2). Compared with the mass spectral data from literature [24,29], elemicin was found to be the unique pure compound isolated from the EOs of the flowers of *D. triradiada*. The compound has a molecular weight of 208.1 g/mol, and the mass spectrum (electron ionization) led to different fragments (Figure 1, Annex). The mass spectra of elemicin (Figure 1) showed a molecular ion peak with *m*/*z* 208 [C_11_H_13_O_3_] + (base peak), the molecular ion was fragmented by losing CH_3_ radical to produce [C_11_H_13_O_3_] + ion at *m*/*z* 193 and this was followed by the most highest peak at *m*/*z* 177, 133, and 77, respectively. The fragmentation pattern was in agreement with that reported by Ekundayo et al. [29].

Alkenylbenzene, elemicin was described in a relatively low percentage in *Deverra tortuosa* (7.3%) [10]. Elemicin (3,4,5-trimethoxyallylbenzene), a fraction of nutmeg oil, is an active natural alkenylbenzene found in many medicinal plants, including several Apiaceae taxa such as *Petroselinum sativum*, *Ferula heuffelii*, *Petroselinum crispum*, etc. [30]. According to the same authors [30], elemicin shows extensive pharmacological effects, including antimicrobial, antioxidants, and anti-acetylcholinesterase [30].

For the EOs of seeds and stems, dillapiol was identified as a major component (82.61 and 82.33%), and apiol was the major compound for the roots’ EOs with 72.16% (Table 2). The essential oils of *D. traradiata* have not been studied before, and to the best of our knowledge, this is the first report profiling the EOs of the species. Among the few taxa belonging to the genus *Deverra* DC (ex *Pituranthos* Viv.), *D. tortuosa* is a well-documented and studied species. Previous studies reported the EOs of *D. tortuosa* in Saudi Arabia [10], Tunisia [31,32], and Egypt [33,34,35]. The authors [10,31,32,33] reported a similar yield of EOs extracted from different parts of *D. tortuosa* not exceeding 0.63 [10]. The chemotype of EOs described herein is different from those described in the literature once compared to closely related species (*D. tortuosa*). Moreover, elemicin, apiol and dillapiol were found to be the major components of *D. triradiata’s* EOs. However, *D. tortuosa’s* EOs was described as a chemotype of apiol for all plant parts in Saudi Arabia [10] as a chemotype of sabinene, α-pinen, limonen, and terpinen-4-ol in Tunisia [29] and as a chemotype of terpinen-4-ol in Egypt [33] and a chemotype of dillapiole, elemicin, and myristicin [34].

### 3.2. Antioxidant Activities as Assessed by EOs’ ABTS System and Plant Extracts’ DPPH System

Table 3A,B show the ABTS values of EOs’ IC_50_ and DPPH values of plant extracts (aerial parts and roots). The values of IC_50_ (ABTS assay system) for EOs are presented in Table 3A. The roots’ EOs and those of stems showed the best antioxidant activity (IC_50_ = 0.282 µg/mL and 0.708 µg/mL, respectively). By comparing the obtained IC_50_ values of *D. triradiata* EOs with that of authentic ascorbic acid (0.413), it was found that the potential antioxidant activity of the EOs is high and not significantly different from the standard. However, the EOs of flowers presented showed a relatively low antioxidant activity (5.42 µg/mL) and were significantly different from ascorbic acid as a standard with the IC_50_ value of 0.413 µg/mL.

For the aerial parts’ extract samples, the results are presented in Table 3B. It is remarkable that in all plant extracts and for three extraction systems (petroleum ether, ethyl acetate, and methanol), IC_50_ values were less than 5 µg/mL. The highest antioxidant activities (DPPH assay system) were observed for the ethyl acetate extracts both for aerial parts and roots (2.47 and 3.18 µg/mL, respectively). The methanol extract of roots and petroleum ether aerial part extract showed the lower values of DPPH scavenging (4.36 µg/mL and 4.52 µg/mL, respectively). IC_50_ values of plant extracts were found to be with high antioxidant potential and are significantly different from ascorbic acid (*p* > 0.05). Moreover, the IC_50_ values of plant extract concentrations were found to be lower by 1.25 to 2 times compared to ascorbic acid concentration for the DPPH system assay (4.36/5.41 = 1.25 times and 2.47/5.41 = 2.19 times).

Our data on antioxidant activity showed values varying between 0.282 and 5.42 µg/mL for the EOs and concentrations ranging between 2.47 and 4.52 µg/mL for plant extracts. Compared to previous published work, antioxidant activity of hydroalcholic extract of *Ferula gummosa* [36] were reported to be moderate, at 579 µg/mL. Plant extracts from *Ferula lutea* showed that the strongest antioxidant activity was obtained for the ethyl acetate extract (IC_50_ = 12.8 µg/mL). However, a recent study focusing on *D. tortuosa* reported relatively comparable results with antioxidant activities for the flowers’ EOS (IC_50_ = 0.175 μg/mL) [10]. According to the same authors, the samples of stems and roots exhibit lower antioxidant activity (IC_50_ = 0.201 μg/mL and 0.182 μg/mL, respectively). Methanol extracts of *D. tortuosa* exhibited the highest antioxidant activities (IC_50_ = 0.064 μg/mL) [10].

This is the first report describing the antioxidant activity of *D. triradiata*. However, the species was described to induce photosensitization [18] and photodermatitis [19]. As a personal observation, we report that the collected plant material of the studied species caused irritations, such as lines on the upper layer of the hand’s skin during collection.

### 3.3. D. triradiata’s EOs Activities against Selected Strains from Malassezia spp. and Candida ssp

**Figure 2:**
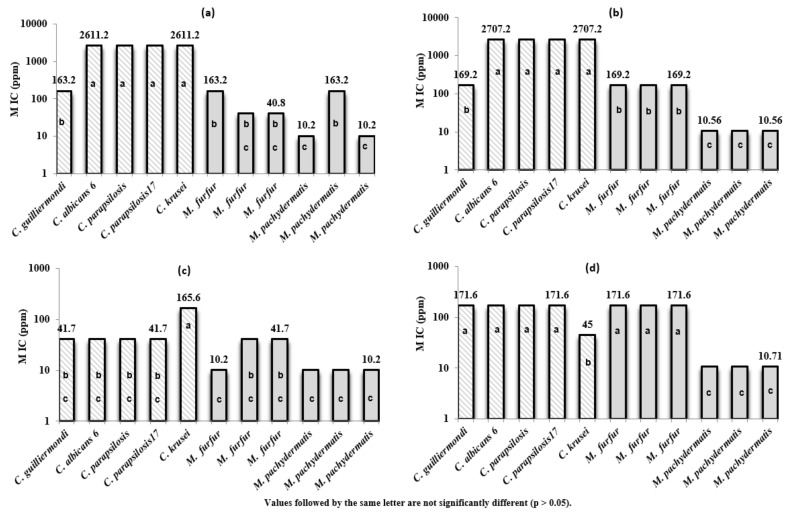
MIC values (ppm) of antifungal activity of the EOs of *D. triradiata*: roots (**a**), seeds (**b**), stems (**c**) and flowers (**d**) against selected strains from two fungus genera *Malassezia* (6 species) and *Candida* (5 species).

The MIC values of 4 EOs (Roots, seeds, stems, and flowers) varied between 3 µL/mL and 48 µL/mL (Table 4 and Figure 2). *M. pachydermatis* (CBS 1978) seems to be the most sensitive fungal strain for the 4 EOs with an MIC value of 3 µL/mL. However, 3 *Candida* strains (*C. albicans* 6, *C. parapsilosis*: ACTT 22020 and *C. parapsilosis*: CD 1378) were found to be with the lowest inhibitory effect against the tested EOs (especially EOs of roots and seeds).

*D. triradiata’s* EOs showed higher inhibitory activities against pathogenic *Malassezia* isolates compared to *Candida* ssp (Figure 2a–d). For *Malassezia* strains, the MIC values of EOs varied between 10.2 and 163.2 ppm for the Roots’ EOs, 10.56 and 169.2 for the seeds’ EOs, 10.2 and 41.7 for the stems’ EOs, and the MIC values of the flowers’ EOs varied from 10.71 to 171.6 ppm. Furthermore, the MIC values of *Candida* species ranged between 163.2 and 2611.2 ppm for Roots’ EOs, 169.2 and 2707.2 for the seeds’ EOs, 41.7 and 165.6 for the stems’ EOs, and 45 to 171.6 for the flowers’ EOs.

**Figure 3:**
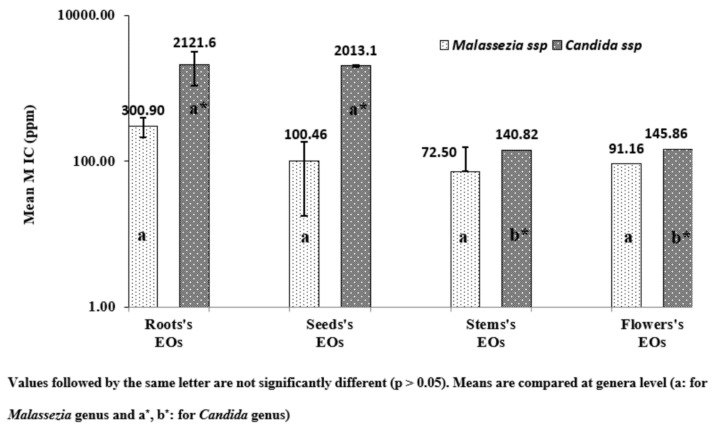
Mean MIC values of antifungal activity of the EOS from *D. triradiata* at genera level against selected strains from two fungus genera *Malassezia* and *Candida*.

The mean MIC values of the two genera are reported in Figure 3. The genus *Malassezia* showed a mean MIC values less than 100 ppm for the 4 tested EOs (only 20.65 ppm for Stems’ EOs). However, the mean MIC values, for candida genus, exceeded 2000 ppm respectively for Roots’ EOs and Seeds’ EOs; the mean MIC values were 66.48 and 146.28 ppm, respectively, for Stems’ EOs and Flowers’ EOs. The oil extracted from the stem was found to be the most potent EOs, with a mean MIC values less than 70 ppm for the two genera. However, pathogenic *Malassezia* and *Candida* isolates seemed to be less sensitive against the oils extracted from the roots and seeds. The chemical profiling of the 4 parts of the plant species (Table 2) showed that the EOs of seeds and stems were reported as a chemotype of dillapiole. This finding could support the effect of minor compounds (mainly seychellene) in the fraction of EOs extracted from the stem which could affect fungal pathogens in synergism with the major components. Moreover, seychellene was reported as a non-selective candidate for inhibitor cyclooxygenase on pre-osteoblast cells [37].

**Figure 4:**
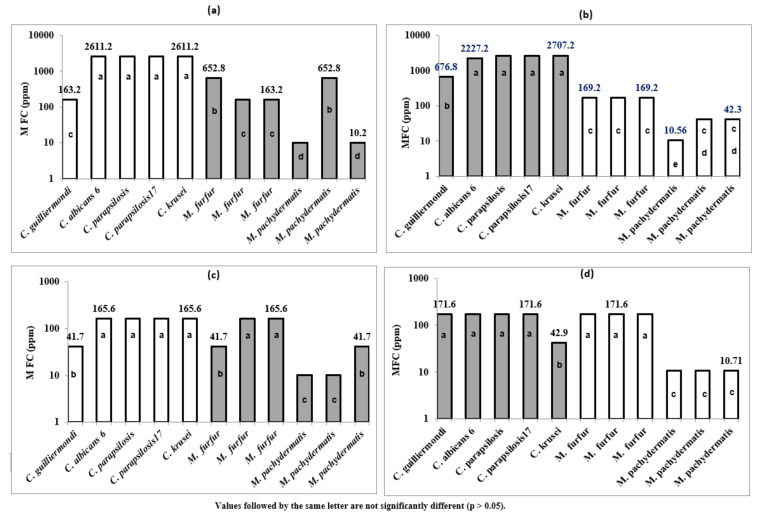
MFC values (ppm) of antifungal activity of the EOs of *D. triradiata*: roots (**a**), seeds (**b**), stems (**c**), and flowers (**d**) against selected strains from two fungus genera *Malassezia* (6 species) and *Candida* (5 species).

Globally, *M. pachydermatis* was the most sensitive yeast to the tested EOs (MIC values ranged from 10.2 and 10.71 ppm). However, *C. parapsilosis*, *C. albicans* 6, and *C. krusei* strains seemed to be the less sensitive, and the highest MIC values were recognized for these three strains (7.15–56.4 mg/mL). The MFC values (Table 4 and Figure 4a–d); Figure 4a–d related to the 11 studied fungal strains corroborated entirely the MIC values for all strains belonging to the 2 genera.

The review of the inhibitory potential of EOs on *Malassezia* and candida species by various plants has revealed the antifungal activity of this valuable natural products extracted from different plant taxa [38,39]. Our data corroborate this finding and the studied EOs exhibited inhibitory potential towards *Candida* and *Malassezia* spp. reference strains. However, the authors [38,39] reported the range of 1000 µg/mL and 2 µL/mL as thresholds to define an EOs as an antifungal inhibitor. Our results showed (in the case of excluding the concentration exceeding 1000 µg/mL) that the tested EOs of stems and flowers of the studied plant species displayed inhibitory potential towards fungal pathogens with concentrations varying between 10.2 to 171.6 ppm and 3 to 12 µL/mL.

Figure 3 showed that EOs possessed fungicidal activity against the tested pathogens belonging to *Candida* and *Malassezia*. The lowest MFC values were recorded against 3 strains of *M. pachydermatis*, and EOs of stems and flowers seem to be the most potent (Figure 4c,d). The finding of the MFC data corroborate those obtained for MIC and *Candida* ssp and were found to be less sensitive, especially towards EOs of roots and seeds. As shown by Figure 3a,b, the highest MFC values (2707.2, 2227.2, and 2611.2 ppm) were recorded against 4 *Candida* strains (*C. albicans* 6, 2 strains of *C. parapsilosis*, and *C. krusei*).

The MFC/MIC ratio was calculated to determine whether thymol has a fungistatic (MFC/MIC ≥ 4) or fungicidal activity (MFC/MIC < 4) [39,40,41]. The MFC/MIC ratio for four studied samples of EOs showed values ranging from to 1 to 2.08. This ratio was equal to 1 for 26 fungal strains out of 44 studied cases (11 fungal strains tested for each of the 4 analysed EOs). In general, the MFC values were found to be lower than the MIC values suggesting that the tested essential oils have fungicidal effects. Our results of MFC described for *D. triradiata* corroborate previous studies focusing on similar fungal strains [10,40,41,42,43]. Guetat et al. (2018) [10] reported that the MFC values of EOs of *D. totuosa* varied between 3 and 24 µL/mL, and *Candida* ssp were found to be less sensitive towards the two tested EOs (extracted from flowers and stems. The relationship between the effectiveness of the studied EOs and the antifungal activity was not discussed by the author [10] and the described chemotype, both for stems and flowers, was reported to be dominated by apiol as a major component. Dongmo et al. [44] reported that the EOs of four Cameroonian spices showed MFC values ranging between 256 and 4096 µL/mL.

### 3.4. Allelopathic Activity of D. triradiata Plant Extracts

The allelopathic effects of the root and aerial parts extracts are summarized in Table S1 (Supplementary Materials). The allelopathic influence on *T. aestivum* L. seeds germination and seedlings growth varied according to the plant extracts and concentration. The germination percentage varied between 65 and 88.23% (values not reported in Table S1). A significant promoting and inhibitory effect on the germination was found (Table S1). The general tendency of the experiment showed that root extracts and aerial part extracts with an inhibitory effect were found to promote germination. The *D. triradiata* ethyl acetate extract of aerial parts showed high phytotoxic effects against *T. aestivum* L. seeds, with an inhibition of seed germination of 17.26% at 0.2 mg/mL. However, the highest induction value of seed germination of the root extracts was exhibited by ethyl acetate extract (12.32% at 0.6 mg/mL).

The variance analysis performed on the allelopathic effects revealed significant differences among concentrations (*p* < 0.05). The effect of the aerial parts’ extracts on the radicle growth varied from 22.73 as an induction factor (at 0.6 mg/mL; ethyl acetate) to −73.4% as inhibition (at 1 mg/mL, petroleum ether). For the root extracts, this character (radicle length) was induced by methanol extract (34.97% at 0.6 mg/mL). However, the radicle length was inhibited by ethyl acetate extract (−9.57% at 0.8 mg/mL).

The hypocotyl length was remarkably induced in the presence of different *D. triradiata* extracts at different concentrations (excepting petroleum ether extract of aerial parts at 1 mg/mL). The highest percentages of promoting were recorded in methanol extracts (from 44.23% at 0.6 mg/mL to 48.86% at 0.4 mg/mL). For the root extracts, the hypocotyl length was highly induced by methanol extracts (57.59% at 1 mg/mL). In this way petroleum ether root extracts also showed high values of hypocotyl length promoting (from 9.61 to 39.31%). The biomass production was considerably induced in the presence of different plant extracts at different concentrations, and the dry weight of the seedlings treated from different samples was highly increased. Globally the root extract increased more remarkably by the dry mass production. For petroleum ether at 1 mg/mL, the highest percentage of dry mass increasing was 66.6% for root extracts and 42.14% for aerial part extracts. The allelopathic effect is related to the plant extract composition and the target species *D. triradiata* extracts was efficient to promote and inhibit the seed germination and the seedling growth of *T. aestivum*. The phytotoxic effect can be mainly due to toxic compounds present in the plant extracts. Secondary plant metabolites such as terpenoids, steroids, phenols, coumarins, flavonoids, tannins, alkaloids, and cyanogenic glycosides, and their degradation products have been known to be involved in allelopathic phenomena [45].

Apiaceae plants are known to accumulate flavonoids mainly in the form of flavonols and flavones [5,46]. The allelochemicals present in *D. triradiata* extracts could, among others, refer to flavonoid metabolites. The action of allelochemicals can affect the respiration, photosynthesis, enzyme activity, water relations, stomatal opening, hormone levels, mineral availability, cell division and elongation, and structure and permeability of cell membranes and walls [46,47,48]. In addition, the allelopathic potential of *D. triradiata* species is not cited in the literature, however, our results corroborate those obtained by Guetat et al. [10] on *D. tortuosa* and by Znati et al. [35] on *Ferula lutea*. The authors [10] reported that the plant extracts of *D. tortuosa* from Saudi Arabia exhibited high phytotoxic effects against *T. aestivum* L. seeds and seedlings. Moreover, the highest germination inhibition (56%) was induced by petroleum ether extracts. The inhibition of the radicle growth was reported to reach 100% (petroleum ether extracts at 1 mg/mL). These findings about the allelopathic potentialities of *D. tortuosa* were supported by Fayed et al. [49]. The authors reported that the EOs showed a substantial allelopathic activity against the weed *Chenopodium murale*. Furthermore, the shoot growth, root growth, and germination was reduced (at higher dose: 100 µL/L) by 84.19, 74.45, and 53.57%, respectively. In addition, the authors ascribed that the EOs of *D. tortuosa* attained IC_50_ values of 94.96, 46.52, and 46.52 μL/L against germination, shoot, and root growth of *C. murale* [49].

## 4. Conclusions

In summary, this study on *D. triradiata* from Saudi Arabia showed that EOs and plant extracts have a variation in their chemical composition. Despite the low chemical diversity of extracted EOs from seeds, roots, stems, and flowers (only nine compounds), the chemicals in the EOs exhibited high biological activity potentials. The highest yield in the essential oils was recorded in stems. Among the tested plant extract samples, the highest antioxidant activity was observed in methanol extracts. Furthermore, the plant extracts also inhibited the shoot and root growth of *Triticum aestivum* seedlings. Moreover, EOs displayed a high inhibitory activity against selected fungal strains (*Malassezia* spp. and *Candida* species). Hence, natural products extracted from *D. triradiata* could be used as a natural herbicide as well as a good source for preventing yeast growth. Our results highlight the value-adding of this Saudi Arabian plant species, which can be a safe and renewable source of biological active compounds for multiple utilizations.

## Figures and Tables

**Table 1:** The used yeast strains in the present study.

Species	Collection Code	Origins
*Candida krusei*	ATCC 6258	American Type Culture Collection
*Candida parapsilosis*	ATCC 22019	American Type Culture Collection
*Candida parapsilosis*	ATCC 22020	American Type Culture Collection
*Candida albicans6*	CD1378	Cloaca of laying hens
*Candida Guilliermondi*	CD1379	Cloaca of laying hens
*Malassezia pachydermatis*	CD90	Dogs with otitis
*Malassezia pachydermatis*	CD 112	Dogs with dermatitis
*Malassezia pachydermatis*	CD 113	Dogs with dermatitis
*Malassezia furfur*	CBS1978	CBS-KNAW Collections
*Malassezia furfur*	CD1029	Human skin
*Malassezia furfur*	CD1006	Human blood

**Table 2:** Chemical composition of four parts of *D. triradiata* Hochst. ex Boiss essential oil from the Northern region of Saudi Arabia.

	Compounds	RI	Roots	Seeds	Stems	Flowers
1	Myristicin	1517	ND	2.03	3.14	ND
2	Seychellene	1458	ND	ND	1.01	ND
3	Dillapiol	1633	ND	82.61	82.33	ND
4	N-[3-(5-methyl-1,3-benzoxazol-2-yl)phenyl]formamide	1672	22.13	11.11	13.52	ND
5	Elemicin	1558	ND	3.12	ND	100
6	(E)-α-elemene	1469	0.84	ND	ND	ND
7	Apiol	1682	72.16	ND	ND	ND
8	7,8,9,10-tetrahydro-anthra[1,2-b]furanne	-	4.68			
9	Germacrene B	1562		1.13		
	Total		99.81%	100%	100%	100%

RI: Retention index relative to n alkanes (C_8_–C_24_) calculated on HP-_5_ capillary column. ND: not detected.

**Table 3:** Antioxidant activity evaluated by ABTS for the EOs (A) and DPPH test assays of plant extracts of *D. triradiata* samples (B).

(A) ABTS IC_50_ (µg/mL)			
**3A.**	**Samples**		**Concentration (µg/mL)**
**EOs**		**Flowers**	5.42 ^a^ (±1.13)
		**Stems**	0.706 ^b^ (±0.14)
		**Roots**	0.282 ^c^ (±0.01)
		**Ascorbic acid**	0.413 ^b,c^ (±0.018)
		(**B) DPPH IC_50_ (µg/µL)**	
**Plant extracts**	**Samples**		**Concentration (µg/mL)**
	**Arial parts**	**Petroleum ether extract**	4.52 ^a^ (±0.95)
		**Ethyl acetate extract**	2.47 ^c^ (±0.2)
		**Methanol extract**	2.73 ^b,c^ (±0.32)
	**Roots**	**Petroleum ether extract**	3.70 ^a,b^ (±0.26)
		**Ethyl acetate extract**	3.18 ^b,c^ (±0.73)
		**Methanol extract**	4.36 ^a^ (±0.1)
		**Ascorbic acid**	5.41 ^a^ (±0.58)

Values are presented as mean ±SD (*n* = 3). Values followed by the same letter are not significantly different (*p* > 0.05).

**Table 4:** Minimum inhibitory concentration (MIC) and minimum fungicidal concentration (MFC) (µg/mL) of *D*. *triradiata* EOS compared to Itraconazole (µg/mL) as a positive control.

	Roots		Seeds		Stems		Flower		Itraconazole	
Fungal Strains	MIC	MFC	MIC	MFC	MIC	MFC	MIC	MFC	MIC	MFC
*C. guilliermondi* (ACTT 6258)	13.6 mg/mL (12 µL/mL)	13.6 mg/mL (12 µL/mL)	14.1 mg/mL (12 µL/mL)	28.2 mg/mL (24 µL/mL)	6.95 mg/mL (6 µL/mL)	6.95 mg/mL (6 µL/mL)	7.15 mg/mL (6 µL/mL)	14.3 mg/mL (12 µL/mL)	32	64
*C. albicans* 6 (ACTT 22019)	54.4 mg/mL (48 µL/mL)	>54.4 mg/mL (48 µL/mL)	56.4 mg/mL (48 µL/mL)	56.4 mg/mL (48 µL/mL)	6.95 mg/mL (6 µL/mL)	13.8 mg/mL (12 µL/mL)	14.3 mg/mL (12 µL/mL)	14.3 mg/mL (12 µL/mL)	> 64	> 64
*C. parapsilosis* (ACTT 22020)	54.4 mg/mL (48 µL/mL)	> 54.4 mg/mL (48 µL/mL)	56.4 mg/mL (48µL/mL)	56.4 mg/mL (48 µL/mL)	6.95 mg/mL (6 µL/mL)	13.8 mg/mL (12 µL/mL)	14.3 mg/mL (12 µL/mL)	14.3 mg/mL (12 µL/mL)	0.32	0.64
*C. parapsilosis*17 (CD 1378)	54.4 mg/mL (48 µL/mL)	> 54.4 mg/mL (48 µL/mL)	56.4 mg/mL (48 µL/mL)	56.4 mg/mL (48 µL/mL)	6.95 mg/mL (6 µL/mL)	13.8 mg/mL (12 µL/mL)	14.3 mg/mL (12 µL/mL)	14.3 mg/mL (12 µL/mL)	0.32	0.64
*C. krusei* (CD 1379)	54.4 mg/mL (48 µL/mL)	> 54.4 mg/mL (48 µL/mL)	56.4 mg/mL (48 µL/mL)	56.4 mg/mL (48 µL/mL)	13.8 mg/mL (12 µL/mL)	13.8 mg/mL (12 µL/mL)	7.15 mg/mL (6 µL/mL)	7.15 mg/mL (6 µL/mL)	0.64	> 0.64
*Candida* ssp.	46.24 mg/mL (± 17.20)	> 46.24 mg/mL (17.20 ±)	47.94 mg/mL (±17.84)	48.76 mg/mL (± 11.54)	8.32 mg/mL (± 2.89)	12.43 mg/mL (± 2.89)	11.44 mg/mL (± 3.69)	12.87 mg/mL (± 3.01)	> 19.54 (± 26.78)	> 25.98 (± 32.72)
*M. furfur* (CD 90)	13.6 mg/mL (12 µL/mL)	27.2 mg/mL (24 µL/mL)	14.10 mg/mL (12 µL/mL)	14.10 mg/mL (12 µL/mL)	3.4 mg/mL (3 µL/mL)	6.95 mg/mL (6 µL/mL)	7.15 mg/mL (6 µL/mL)	14.3 mg/mL (12 µL/mL)	0.32	0.32
*M. furfur* (CD 112)	6.8 mg/mL (6 µL/mL)	13.6 mg/mL (12 µL)	14.10 mg/mL (12 µL/mL)	14.10 mg/mL (12 µL/mL)	6.95 mg/mL (6 µL/mL)	13.8 mg/mL (12 µL/mL)	7.15 mg/mL (6 µL/mL)	14.3 mg/mL (12 µL/mL)	0.32	0.32
*M. furfur*(CD 113)	6.8 mg/mL (6 µL/mL)	13.6 mg/mL (12 µL/mL)	14.10 mg/mL (12 µL)	14.10 mg/mL (12 µL/mL)	6.95 mg/mL (6 µL/mL)	13.8 mg/mL (12 µL/mL)	7.15 mg/mL (6 µL/mL)	14.3 mg/mL (12 µL/mL)	0.32	0.32
*M. pachydermatis* (CBS 1978)	3.4 mg/mL (3 µL/mL)	3.4 mg/mL (3 µL/mL)	3.52 mg/mL (3 µL/mL)	3.52 mg/mL (3 µL/mL)	3.4 mg/mL (3 µL/mL)	3.4 mg/mL (3 µL/mL)	3.57 mg/mL (3 µL)	3.57 mg/mL (3 µL/mL)	0.32	0.32
*M. pachydermatis* (CD 1029)	13.6 mg/mL (12 µL/mL)	27.2 mg/mL (24 µL/mL)	3.52 mg/mL (3 µL/mL)	7.05 mg/mL (6 µL/mL)	3.4 mg/mL (3 µL/mL)	3.4 mg/mL (3 µL/mL)	3.57 mg/mL (3 µL/mL)	3.57 mg/mL (3 µL/mL)	0.32	0.32
*M. pachydermatis* (CD 1006)	3.4 mg/mL (3 µL/mL)	3.4 mg/mL (3 µL/mL)	3.52 mg/mL (3 µL/mL)	7.05 mg/mL (6 µL/mL)	3.4 mg/mL (3 µL/mL)	6.95 mg/mL (6 µL/mL)	3.57 mg/mL (3 µL/mL)	3.57 mg/mL (3 µL/mL)	0.32	0.32
*Malassezia* ssp.	7.93 mg/mL (±4.43)	14.73 mg/mL (±10.18)	8.81 mg/mL (±5.53)	9.99 mg/mL (±4.47)	4.58 mg/mL (±1.75)	8.05 mg/mL (±4.51)	5.36 mg/mL (±1.87)	8.94 mg/mL (±5.6)	0.32 (±0.00)	0.32 (±0.00)

## Data Availability

Not applicable.

## Supplementary Materials

The following supporting information can be downloaded at: https://www.mdpi.com/article/10.3390/plants11121543/s1, Table S1: Allelopathic effects of the plant extracts of *D. triradiata* samples at different concentrations on *Triticum aestivum* L. seedlings.
