# Supplementary material for: Deverra triradiata Hochst. ex Boiss. from the Northern Region of Saudi Arabia: Essential Oil Profiling, Plant Extracts and Biological Activities
Source: Plants (Basel). 2022 Jun 9;11(12):1543. doi: 10.3390/plants11121543 (PMC9231278; doi:10.3390/plants11121543)
Supplement: Supplementary file 1 [file plants-11-01543-s001.zip › plants-1718269-supplementary.pdf]

Table S1 Allelopathic effects of the plant extracts of *D. triradiata* samples at different concentrations on *Triticum æstivum* L.

(a) **Inhibitory effect compared to control (%)**

Plant extract concentration (mg/ml)		0.2			0.4			0.6			0.8			1		
		**Pet.Et**	**Et.Ac**	**Met**	**P.Et**	**Et.Ac**	**Met**	**P.Et**	**Et.Ac**	**Met**	**P.Et**	**Et.Ac**	**Met**	**P.Et**	**Et.Ac**	**Met**
**Aerial parts extracts**	**Seed germination**	-1^{g}	-17.26^{n}	-15.14^{m}	9.11^{b}	-2.86^{i}	-8.23^{k}	0.49^{f}	6.08^{c,d}	11.38^{a}	3.84^{e}	5.56^{d}	-3.76^{j}	-10.55^{l}	6.65^{c}	-2.08^{h}
	**Radicle length**	1.19^{d}	-2.52^{g}	-8.64^{h}	22.48^{a}	7.57^{c}	7.97^{c}	-51.46^{l}	22.73^{a}	16.22^{b}	-0.53^{f}	5.71^{d}	-16.22^{i}	-73.40^{m}	-25.13^{k}	-20.74^{j}
	**Hypocotyl length**	17.94^{k}	42.09^{d}	34.40^{g}	21.58^{j}	40.81^{e}	47.86^{a}	3.63^{l}	38.88	44.23^{c}	31.19^{h}	39.74^{e,f}	30.34^{h}	-38.24^{m}	23.29^{i}	45.72^{b}
	**Seedling dry weight**	42.14^{a}	4.95^{h}	-25.61^{l}	7.43^{f}	0^{j}	5.78^{g,h}	1.65^{i}	9.91^{e}	6.61^{f,g}	11.57^{d}	33.05^{b}	7.43^{f}	-16.52^{k}	23.14^{c}	0^{j}

(b) **Inhibitory effect compared to control (%)**

Plant extract concentration (mg/ml)		0.2			0.4			0.6			0.8			1		
		**Pet.Et**	**Et.Ac**	**Met**	**P.Et**	**Et.Ac**	**Met**	**P.Et**	**Et.Ac**	**Met**	**P.Et**	**Et.Ac**	**Met**	**P.Et**	**Et.Ac**	**Met**
**Roots extracts**	**Seed germination**	10.54^{c}	4.38^{e}	5.56^{d}	-3.26^{i}	1.36^{g}	10.54^{c}	-1.80^{h}	12.32^{a}	11.04^{c}	3.21^{f}	-7.18^{j}	3.61^{e,f}	12.02^{a,b}	-3.26^{i}	11.38^{b,c}
	**Radicle length**	-1.72^{h}	12.5^{e}	16.88^{d}	-3.45^{i}	-8.37^{j}	9.44^{f}	1.72^{g}	22.2^{c}	34.97^{a}	-2.26^{h}	-9.57^{k}	24.06^{b}	15.95^{d}	-4.25^{i}	23.67^{b}
	**Hypocotyl length**	9.61^{l}	33.97^{f}	43.58^{c}	36.75^{e}	22.22^{j}	30.34^{g}	39.31^{d}	26.28^{h}	43.58^{c}	25.21^{h,i}	19.44^{k}	46.79^{b}	24.78^{i}	20.72^{k}	57.59^{a}
	**Seedling dry weight**	28.92^{e}	20.66^{g}	33.05^{d}	14.87^{j}	52.06^{b}	16.52^{i}	8.26^{m}	18.18^{h}	44.62^{c}	1.65^{n}	11.57^{l}	21.48^{g}	66.11^{a}	24.79^{f}	13.22^{k}

seedlings.a) Aerial parts Extracts; b) Roots extracts; Pet Et: Petroleum Ether, Et Ac: Ethyl Acetate and Met: Methanol. Inhibitory effect (%) is calculated as follows: PytPot *D. triradiata* (%) = [(PEE– Ce) /CE] ×100; Where PEE: the effect of plant extract and CE : the null effect of the negative control. Values in line not sharing common letters are significantly different ($p < 0.05$).
